# Clarification on the name-bearing type designation of several cyclophorid species (Mollusca, Gastropoda) by H. H. Godwin-Austen (1915)

**DOI:** 10.3897/zookeys.1049.66842

**Published:** 2021-07-16

**Authors:** Parin Jirapatrasilp, Jonathan D. Ablett, Somsak Panha, Chirasak Sutcharit

**Affiliations:** 1 Animal Systematics Research Unit, Department of Biology, Faculty of Science, Chulalongkorn University, Bangkok 10330, Thailand Chulalongkorn University Bangkok Thailand; 2 Department of Life Sciences, Natural History Museum, London, SW7 5BD, UK Natural History Museum London United Kingdom; 3 Academy of Science, The Royal Society of Thailand, Bangkok 10300, Thailand Academy of Science, The Royal Society of Thailand Bangkok Thailand

**Keywords:** Cyclophoridae, hypodigm, ICZN, NHM, nomenclature, NZSI, onomatophore

## Abstract

The type series boundary and the name-bearing type designation of each cyclophorid taxon originally described by Godwin-Austen are clarified based on an interpretation that complies with the ICZN. Previous statuses of type specimens designated by previous authors are reconsidered. Lectotypes of *Spiraculum oakesi* Godwin-Austen, 1915, *Spiraculum kempi* Godwin-Austen, 1915, *Pterocyclos aborensis* Godwin-Austen, 1915, *Pterocyclos miriensis* Godwin-Austen, 1915, *Pterocyclos brahmakundensis* Godwin-Austen, 1915, *Spiraculum luyorensis* Godwin-Austen, 1915, *Spiraculum putaoensis* Godwin-Austen, 1915, and *Theobaldius oakesi* Godwin-Austen, 1915 are here designated to stabilize the existing nomenclature. In addition, the type specimens of *Pterocyclos miriensis* and *Theobaldius oakesi* are photographed and figured for the first time.

## Introduction

The phylogenetic analyses of the operculated land snail genus *Cyclophorus* (Caenogastropoda: Cyclophoridae) from Thailand uncovered a high degree of intra- and interspecific morphological variation and a wide distribution of the genus (Nantarat et al. 2014b, c; 2019). Southeast Asia, including Thailand, also hosts a high diversity of related cyclophorid genera, such as *Pterocyclos* Benson, 1832, *Spiraculum* Pearson, 1833 (= *Pearsonia* Kobelt, 1902), and *Rhiostoma* Benson, 1860, in which the members of each genus are conchologically very similar (BEDO 2017; Sutcharit et al. 2018), and for which precise species identification is not possible without direct comparison with the type specimens.

The Natural History Museum in London (hereafter the NHM) holds the type specimens of 42 nominal *Cyclophorus* species (Nantarat et al. 2014a), which is approximately a quarter of all currently recognized species (Kobelt 1902, 1908). The type specimens of 95 nominal species in six other cyclophorid genera, namely *Crossopoma* Martens, 1891, *Cyclotus* Swainson, 1840, *Myxostoma* Troschel, 1847, *Pterocyclos*, *Scabrina* Blanford, 1863, *Spiraculum*, and *Rhiostoma* are also housed in the NHM (Sutcharit et al. 2019), and constitute about half of all currently known nominal species of these genera (Kobelt 1902). These type specimens have already been catalogued and illustrated, and in certain cases lectotypes were designated in accordance with the International Code of Zoological Nomenclature (ICZN 1999) to stabilize the usage of each nominal name (Nantarat et al. 2014a; Sutcharit et al. 2019).

Of the type specimens housed in the NHM, the cyclophorid taxa originally described in the “Zoological Results of the Abor Expedition” by Godwin-Austen (1915) require special consideration as the original descriptions contain the explicit designation of “Type” and specimen lot numbers (which correspond to the NHMUK registration numbers; note: NHM is the institutional acronym, whilst NHMUK is the registration number prefix of samples kept at the NHM). This way of type designation was not applied in the other works of Godwin-Austen in the same series (Godwin-Austen 1914b, 1916, 1917, 1918a, b). In contrast, the type lot numbers were explicitly designated in the original descriptions of some taxa in other monographs by Godwin-Austen (1910, 1914a, 1920). Consequently, the interpretation of Godwin-Austen’s type series boundaries and the designation of the name-bearing type(s) is often contentious (Nantarat et al. 2014a; Sajan et al. 2019).

In this article, we review the type status of all cyclophorid taxa originally described by Godwin-Austen (1915). Some lectotype designations by Nantarat et al. (2014a) and Sutcharit et al. (2019) and the type status of *Cyclophorus koboensis* as recognized by Sajan et al. (2019) are reconsidered as we clarify the boundaries of the type series and the name-bearing type designation of each taxon.

## The problem with the name-bearing type designations in Godwin-Austen (1915)

When Godwin-Austen (1915) introduced new taxa, he explicitly placed the word “Type” in the beginning of the first paragraph below the shell description and dimensions (hereafter regarded as the “Type” paragraph), and this was followed by the specimen lot number belonging to either “Ind. Mus.”, currently The National Zoological Collection of the Zoological Survey of India (hereafter the NZSI), or “B.M.” referring to the ‘British Museum (Natural History)’ as it was then known (currently the NHM). However, the original descriptions of some taxa contained more than one specimen lot number, either separated by a semicolon in the same paragraph or appearing in subsequent paragraphs. In addition, for some taxa Godwin-Austen (1915) provided figures of more than one specimen from different specimen lots, but associated the word “Type” in the plate caption to the figures of one specimen only. Therefore, to clarify the name-bearing type designation by Godwin-Austen (1915), it is necessary to answer the following two questions, with verbatim applicable definitions and articles from the fourth edition of the ICZN online version (ICZN 1999) provided in italics:

### 1) Which specimens constitute the type series as recognized by Godwin-Austen (1915)?

*Article 72.4.1. The type series of a nominal species-group taxon consists of all the specimens included by the author in the new nominal taxon (whether directly or by bibliographic reference), except any that the author expressly excludes from the type series [Art. 72.4.6], or refers to as distinct variants (e.g. by name, letter or number), or doubtfully attributes to the taxon.*

*Article 72.4.1.1. For a nominal species or subspecies established before 2000, any evidence, published or unpublished, may be taken into account to determine what specimens constitute the type series.*

*Article 72.4.6. If an author when establishing a nominal species-group taxon nominates either “syntypes” (by that term, or by use of one of the terms “cotypes” or “types” alone), or “holotype and paratypes” used together (or by use of the term “type” together with “allotype” or “cotypes”), and also lists other specimens, the separate mention of the latter expressly excludes them from the type series.*

*Article 72.4.7. The mere citation of “Type” or equivalent expression, in a published work other than that in which the nominal species-group taxon is established, or in an unpublished catalogue of a museum, or on a label, is not necessarily evidence that a specimen is or is fixed as any of the kinds of types referred to in this Chapter.*

*Article 73.2. Syntypes are specimens of a type series that collectively constitute the name-bearing type. They may have been expressly designated as syntypes (see Article 73.2.1 for acceptable terms); for a nominal species-group taxon established before 2000 [Art. 72.3] all the specimens of the type series are automatically syntypes if neither a holotype [Art. 72.1] nor a lectotype [Art. 74] has been fixed. When a nominal species-group taxon has syntypes, all have equal status in nomenclature as components of the name-bearing type.*

Godwin-Austen (1915) did not explicitly indicate which specimens were included or excluded from the type series, as he did not use terms such as “syntypes”, “cotypes”, “types”, “type and cotypes” or “holotype and paratypes”, while he did refer to other specimens, so that the actual type series cannot be unequivocally delimited using Art. 72.4.6. Therefore, based on Art. 72.4.1., the type series of each taxon recognized by Godwin-Austen (1915) should consist of all specimen lots mentioned in the original description, except any that the author referred to as distinct variants.

**Figure 1.**
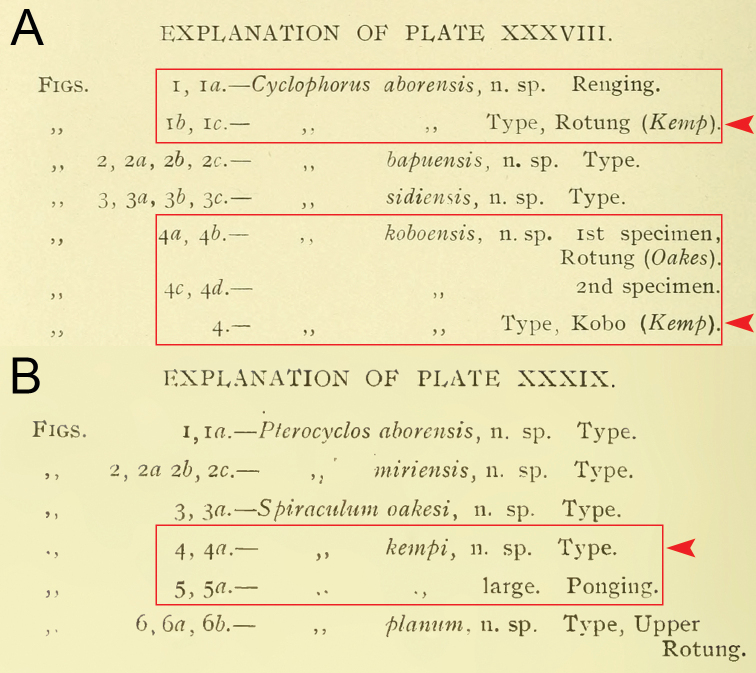
Figure caption with no page number of some cyclophorid taxa originally described by Godwin-Austen (1915) in **A** plate 38 and **B** plate 39. Red boxes indicate the figure caption of the same taxa. Red arrows indicate the annotation of “Type”. Credit: The Biodiversity Heritage Library.

**Figure 2.**
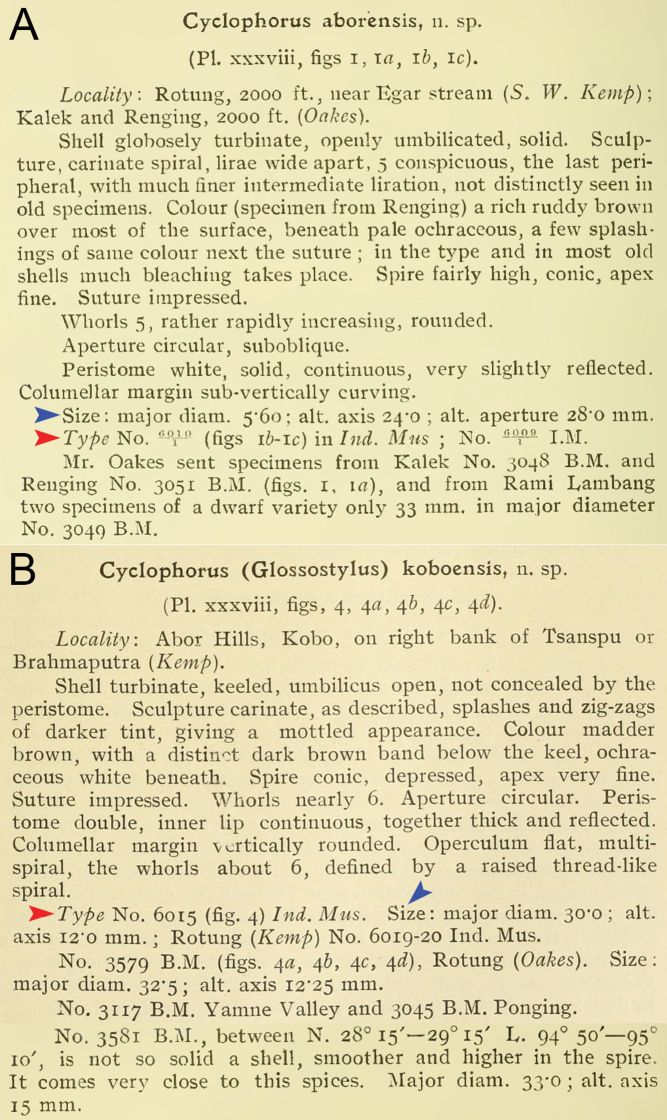
Original descriptions of **A***Cyclophorus aborensis* and **B**Cyclophorus (Glossostylus) koboensis from Godwin-Austen (1915). Red arrows indicate the annotation of “Type”. Blue arrows indicate the set of measurements. Credit: The Biodiversity Heritage Library.

**Figure 3.**
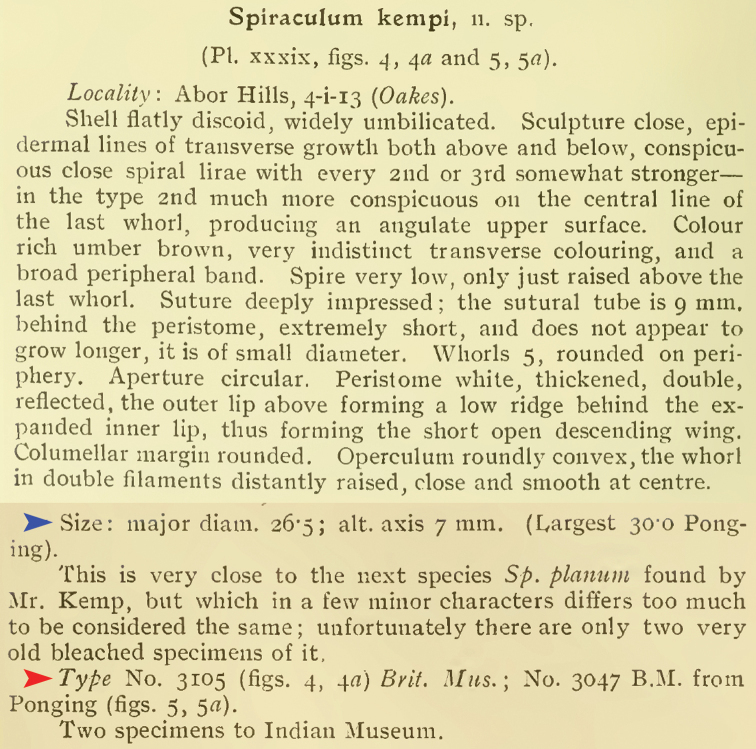
Original description of *Spiraculum kempi* from Godwin-Austen (1915). Red arrow indicates the annotation of “Type”. Blue arrow indicates the set of measurements. Credit: The Biodiversity Heritage Library.

**Figure 4.**
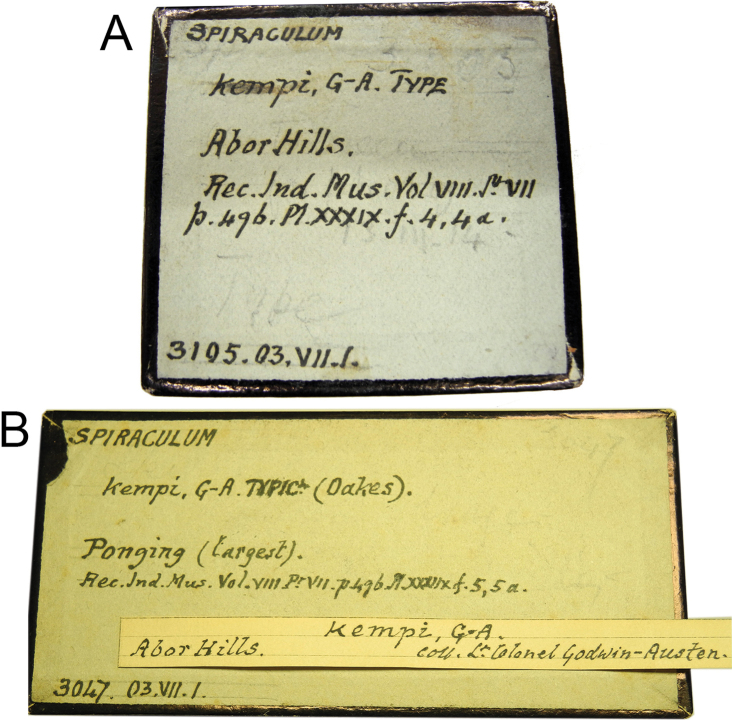
Original labels bearing Godwin-Austen’s handwriting of *Spiraculum kempi***A** NHMUK 1903.7.1.3105 with the word “Type” and **B** NHMUK 1903.7.1.3047 with the word “Typic.”.

**Figure 5.**
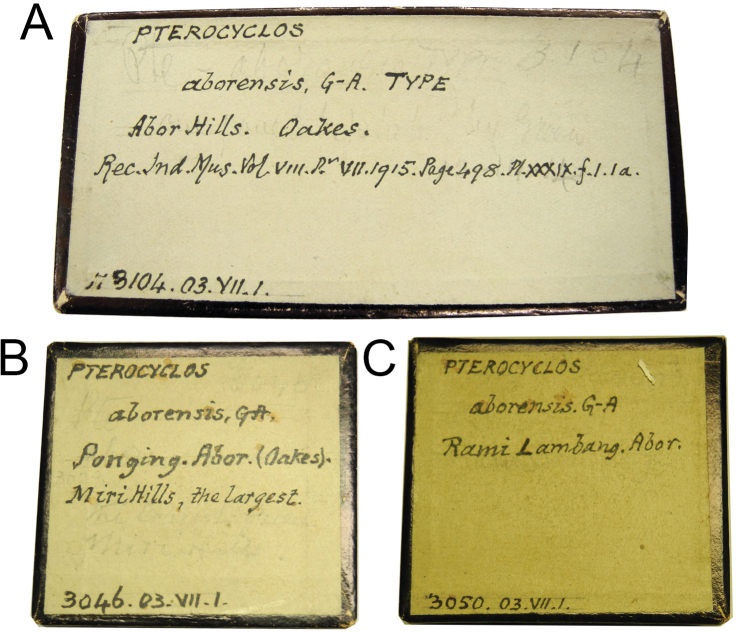
Original labels bearing Godwin-Austen’s handwriting of *Pterocyclos aborensis***A** NHMUK 1903.7.1.3104 with the word “Type” **B** NHMUK 1903.7.1.3046, and **C** NHMUK 1903.7.1.3050.

**Figure 6.**
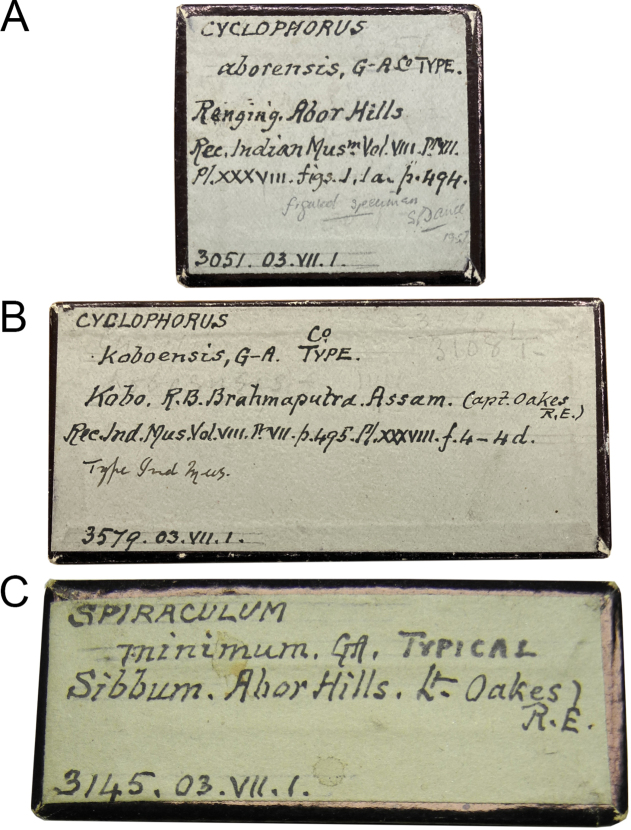
Original labels bearing Godwin-Austen’s handwriting of **A***Cyclophorus aborensis* NHMUK 1903.7.1.3051 with the word “Co-Type” **B**Cyclophorus (Glossostylus) koboensis NHMUK 1903.7.1.3579 with the word “Co-Type”, and **C***Spiraculum minimum* NHMUK 1903.7.1.3145 with the word “Typical” in different handwriting.

According to Art. 72.4.1.1, additional evidence found within or outside the original descriptions, either published or unpublished, may be considered when determining which specimens constitute a type series. Yet, if we do so for the type material of Godwin-Austen (1915), then we are confronted with the following situation. On the one hand, for taxa of which more than one specimen from multiple specimen lots were illustrated, each specimen which was marked as “Type” in the plate captions (Fig. 1) always belongs to the first lot in the “Type” paragraph (Figs 2, 3) and this first specimen lot was always labelled as “Type” in Godwin-Austen’s handwriting (Figs 4A, 5A and Sajan et al. 2019: fig. 1h). On the other hand, specimens from other specimen lots, either in the text delimited from the first lot by a semi-colon or mentioned in subsequent paragraphs, are never marked as “Type” in the plate captions (Fig. 1) or elsewhere in the text. Likewise, these other specimen lots were never labelled as “Type” (Fig. 5B, C), although they may be marked as “Co-Type”, “Typic”, or “Typical” in Godwin-Austen’s handwriting (Figs 4B, 6). According to Art. 72.4.7., the mere citation of “Type” or its equivalent expression on a label does not by itself indicate that those specimens are fixed as any of the kinds of types. In addition, the labels “Typic” and “Typical” did not always relate to type material in the current sense of the word (see Raheem et al. 2014). Therefore, one can argue to restrict the type series of Godwin-Austen’s (1915) taxa to the first and only specimen lot in the “Type” paragraph.

These two possible interpretations of the type series are rooted in the ambiguous usage of the “type” terminology in the 19^th^ century. The term “type” had been given three operational functions by Simpson (1940) as: “(a) a sample from which the characters of a group of individuals or a population are estimated, (b) a standard of comparison between samples, or (c) name-bearers” (Melville 1970). The first two are taxonomic functions of type, while the third one is the nomenclatural function (Dubois 2005). As such, Simpson (1940) introduced the term “hypodigm” for the first two taxonomic functions, meaning “all the specimens used by the author of a species as his basis for inference, and this should mean all the specimens that he referred to the species, constitute his hypodigm of that species”. For the third function of “types”, i.e., as name-bearers, several terms were proposed, two of which, “onomatophore” (Simpson 1940) and “nomenifer” (Schopf 1960), have been more frequently adopted (e.g., Dubois 2005; Sluys 2021). Here we will use the term “onomatophore” to refer to the name-bearer simply because this term was introduced first.

The ICZN regulates the nomenclatural rules but is not involved in “restricting the freedom of taxonomic thought or actions” (ICZN 1999). Hence, the ICZN is supposed to only deal with rules regulating onomatophores, not with rules that regulate the function of “types” as “hypodigm”. However, in Art. 72.4.1. the definition of the type series is identical to that of Simpson’s (1940) hypodigm. Consequently, according to Art. 73.2., for all taxa originally described before 2000 and for which neither a holotype, nor a lectotype has been fixed, the Code automatically equates the original hypodigm (= all specimens in the type series) with onomatophores (= syntypes). So, the application of this article is problematic because the word “type” in the term “type series” does not have the same function as in the term “name-bearing type”, as was recognized earlier (Melville 1970). This misunderstanding that the name-bearing type possesses taxonomic functions, in being “a typical example, a prototype, or an archetype of the species to which it belongs and to which it affixes a name” still prevails to this day (Sluys 2021). See Witteveen (2016) for more details on the development of the type concept in both taxonomic and nomenclatural functions.

The problem of defining Godwin-Austen’s (1915) type series arises because we posit that Godwin-Austen (1915) assigned specimens to the original hypodigm and onomatophores differently. The type series, as defined by Art. 72.4.1., corresponds well to the hypodigm concept. Accordingly, Godwin-Austen (1915) in establishing the original hypodigm applied the terms “Typic” or “Typical” on the label of some specimen lots mentioned in the original description. However, the term “Type” in the sense of onomatophore, as recognized by Godwin-Austen (1915), cannot apply to all specimens in the type series because accepting all specimens in the type series as types would contradict the writing structure of Godwin-Austen’s (1915) original descriptions, plate captions, and the labels of the specimens. As such, the onomatophores as recognized by Godwin-Austen (1915) are limited to the first and only specimen lot in the “Type” paragraphs and corresponding to the labels in Godwin-Austen’s handwriting of the respective specimen lots.

Godwin-Austen’s (1915) interpretative “type” problem is illustrated by *Spiraculum kempi*. Two specimen lots, “No. 3105 B.M.” from Abor Hills and “No. 3047 B.M.” from Ponging, were mentioned in the original description so these two lots constitute the original hypodigm and become the type series. However, only the specimen from lot “No. 3105 Brit. Mus.” was marked as “Type” in the plate caption (Fig. 1B) and this lot was labelled as “Type” in Godwin-Austen’s handwriting (Fig. 4A). In contrast, specimen lot “No. 3047 B.M.” was not marked as “Type” in the plate caption and was labelled as “Typic.” in Godwin-Austen’s handwriting (Fig. 4B). Thus, this could mean that Godwin-Austen (1915) designated only lot “No. 3105 B.M.” as onomatophores, but not lot “No. 3047 B.M.”. However, without an explicit holotype designation or any equivalent expression (see below), the Code dictates that all specimens in both lots constitute the type series and so automatically become syntypes.

Consequently, following Art. 72.4.1. the type series cannot be restricted to only the first lot in the type paragraph, and this action corresponds well to the hypodigm as recognized by Godwin-Austen (1915). However, as the Code automatically equates the original hypodigm to onomatophores if there is no holotype designation, it is possible that a subsequent author selects a lectotype from a syntype that is not part of the originally intended onomatophores, but that is part of the original hypodigm. This has happened with *Spiraculum minimum* when Sutcharit et al. (2019) designated a specimen that was not part of the originally intended onomatophores as lectotype (see below), an action that is deemed valid under the Code.

### 2) Are there indications in the holotype designation that comply with an “equivalent expression”?

*Article 73.1.1. If an author when establishing a new nominal species-group taxon states in the original publication that one specimen, and only one, is the holotype, or “the type”, or uses some equivalent expression, that specimen is the holotype fixed by original designation.*

*Article 73.1.2. If the nominal species-group taxon is based on a single specimen, either so stated or implied in the original publication, that specimen is the holotype fixed by monotypy (see Recommendation 73F). If the taxon was established before 2000 evidence derived from outside the work itself may be taken into account [Art. 72.4.1.1] to help identify the specimen.*

*Recommendation 73F. Avoidance of assumption of holotype. Where no holotype or syntype was fixed for a nominal species-group taxon established before 2000, and when it is possible that the nominal species-group taxon was based on more than one specimen, an author should proceed as though syntypes may exist and, where appropriate, should designate a lectotype rather than assume a holotype (see also Article 74.6).*

The indication whether the holotype designation of each taxon comply with an “equivalent expression” is a crucial point because if the original author explicitly designated only one onomatophore (= holotype), that holotype designation would comply with the Code and there would be no contradiction in accepting a type (series) as the original hypodigm. Unfortunately, Godwin-Austen (1915) used the term “Type” instead of “Holotype”, and although the term “holotype” had already been coined in the late 19^th^ century (Schuchert 1897), it was not commonly used until the 1950s. For example, Simpson (1940) and Newell (1949) still used the term “Type”, although they mentioned “Holotype” in their works, whereas Shenefelt (1959), Schopf (1960), and Simpson (1960) did apply the term “Holotype”. However, in some taxa Godwin-Austen (1915) additionally referred to “the type” in the body of texts. It is, therefore, necessary to demonstrate whether Godwin-Austen (1915) applied “the type” in the sense of a holotype or as an equivalent expression of a holotype designation.

We identified four ways of type annotation by Godwin-Austen (1915). The first way is the most prevalent among his original descriptions, i.e., those for which multiple specimen lots are mentioned in the description, whereas only one specimen is figured and marked as “Type” in the plate caption. This way of type annotation is not an equivalent expression of a holotype designation, as it can refer to any “Type” rather than specifically to “Holotype”, while it does not unequivocally imply a single specimen. This interpretation is similar to that of some taxa listed by Cowie et al. (2017) (e.g., *fraternus* Pilsbry & Bequaert, 1927). In addition, parallel to a lectotype designation before 2000 (ICZN 1999: Arts. 74.5., 74.6.), the expression “Type” does not a priori demonstrate that an author regarded a given specimen as a unique, name-bearing type, and simply figuring a specimen with a “Type” caption is not enough to change this (Welter-Schultes 2013; Calhoun 2017). Therefore, in such cases all specimens in the type series are syntypes and all have an equal nomenclatural status as name-bearing types.

The second way of type annotation by Godwin-Austen (1915) occurs in the original description of *Spiraculum kempi*, which mentions two specimen lots (Fig. 3). Two specimens, each from a different lot, were figured but only one specimen was marked as “Type” in the plate caption (Fig. 1B). Although this way of type annotation is more specific in pinpointing a single specimen of a particular lot, the expression “Type” still does not necessarily indicate a unique, name-bearing type selected by the original author. Moreover, the specimen lot to which the specimen marked as “Type” in the plate caption belongs, contains more than one specimen. Thus, following Recommendation 73F to avoid the assumption of a holotype, we regard all the specimens in the type series as syntypes.

The third way of Godwin-Austen’s (1915) type annotation occurs in *Cyclphorus koboensis* and is an extension of the second way, in that a set of shell measurements was added to each specimen lot number in the “Type” paragraph (and subsequent paragraphs) (Fig. 2B). In all the other taxa, these measurements were provided in their own lines above the “Type” paragraph (e.g., Figs 2A, 3). The “Type” annotation in *C. koboensis* is an equivalent expression of a holotype designation similar to some cases in Cowie et al. (2017) (e.g., *langi* Pilsbry & Bequaert, 1927 and *planogyra* Pilsbry, 1933).

Finally, the fourth way of type annotation occurs in the original description of *Cyclophorus aborensis*. Two specimens were figured in the plate but only one specimen was marked as “Type” in the plate caption (Fig. 1A), and this specimen belongs to the specimen lot number with the number one under the horizontal fraction bar (Fig. 2A). This is an equivalent expression of a holotype designation as the number under the horizontal fraction bar is commonly used to represent the number of the specimen in that lot, and this means that only one specimen was designated as the type by Godwin-Austen (1915).

Although Godwin-Austen (1915) referred to “the type” in the body of the text of the original descriptions of *C. aborensis* and *S. kempi*, this should not be taken as implying only one single individual. It is more likely that “the type” in Godwin-Austen’s sense indicated an association to one specimen lot, which sometimes contains more than one specimen (e.g., *S. kempi*).

## Status of Godwin-Austen’s (1915) cyclophorid taxon name-bearing type(s)

The order of taxa below follows that of Godwin-Austen (1915) and the recent species combination follows MolluscaBase (2021). A summary is given in Table 1.

**Table 1.** Current status of type series, other non-type materials, type locality, and original onomatophores of cyclophorid taxa in Godwin-Austen (1915). The number of shells in some specimen lots are unknown and not specified.

Taxon	Type series	Type locality	Original onomatophores recognized by Godwin-Austen (1915)	Remarks
1. *Cyclophorus aborensis*	Holotype NZSI M.6010/1. Paratypes NZSI M.6009/1, NHMUK 1903.7.1.3048, NHMUK 1903.7.1.3051	Rotung, 2000 ft., near Egar stream	NZSI “No. 6010/1” (1 shell)	Invalid lectotype and paralectotype designation by Nantarat et al. (2014a).
2. Cyclophorus (Glossostylus) bapuensis	Lectotype NHMUK 1903.7.1.3108/1. Paralectotypes NHMUK 1903.7.1.3108/2–3	Abor Hills, vicinity of Bapu	NHMUK 1903.7.1.3108 (4 shells)	Valid lectotype and paralectotype designation by Nantarat et al. (2014a).
3. Cyclophorus (Glossostylus) sidiensis	Syntypes NZSI M.6002, NZSI M.6001, NHMUK 1903.7.1.3095	On Sidi River, Abor Hills; Rotung; Tsanpu Valley	NZSI “No. 6002”	–
4. Cyclophorus (Glossostylus) koboensis	Holotype NZSI M.6015/1. Paratypes NZSI M.6019–20, NHMUK 1903.7.1.3045, NHMUK 1903.7.1.3117, NHMUK 1903.7.1.3579	Abor Hills, Kobo, on right bank of Tsanspu or Brahmaputra River	NZSI “No. 6015” (1 shell)	Invalid lectotype and paralectotype designation by Nantarat et al. (2014a).
5. *Spiraculum oakesi*	Lectotype (design. nov.) NHMUK 1903.7.1.3081/1. Paralectotypes NHMUK 1903.7.1.3081/2–5, NZSI	Abor Hills	NHMUK 1903.7.1.3081 (5 shells)	–
6. *Spiraculum kempi*	Lectotype (design. nov.) NHMUK 1903.7.1.3105/1. Paralectotypes NHMUK 1903.7.1.3105/2, NHMUK 1903.7.1.3047, NZSI	Abor Hills	NHMUK 1903.7.1.3105 (2 shells)	–
7. *Spiraculum planum*	Syntypes NZSI M.5992, NZSI M.5992a	Upper Rotung, Abor Hills; Yembung	NZSI “No. 5992”	–
8. *Pterocyclos aborensis*	Lectotype (design. nov.) NHMUK 1903.7.1.3104/1. Paralectotypes NHMUK 1903.7.1.3104/2–3, NHMUK 1903.7.1.3046, NHMUK 1903.7.1.3050	Abor Hills	NHMUK 1903.7.1.3104 (3 shells)	–
9. *Pterocyclos miriensis*	Lectotype (design. nov.) NHMUK 1903.7.1.3580/1 (Fig. 8A).	Miri Hills	NHMUK 1903.7.1.3580 (4 shells)	–
	Paralectotypes NHMUK 1903.7.1.3580/2–4			
10. *Pterocyclos spiramentum*	Holotype NHMUK 1903.7.1.3082	Abor Hills	NHMUK 1903.7.1.3082 (1 shell)	–
11. *Pterocyclos brahmakundensis*	Lectotype (design. nov.) NHMUK 1903.7.1.713/1. Paralectotypes NHMUK 1903.7.1.713/2–3	Brahmakund, Eastern Assam	NHMUK 1903.7.1.713 (3 shells)	–
12. *Spiraculum luyorensis*	Lectotype (design. nov.) NHMUK 1903.7.1.3530/1	Luyor, Abor Hills	NHMUK 1903.7.1.3530 (1 shell)	–
13. *Spiraculum putaoensis*	Lectotype (design. nov.) NHMUK 1903.7.1.3598/1. Paralectotypes NHMUK 1903.7.1.3598/2–3	Putao, Upper Burma	NHMUK 1903.7.1.3598 (3 shells)	–
14. *Spiraculum minimum*	Lectotype NHMUK 1903.7.1.3145/1. Paralectotypes NHMUK 1903.7.1.3145/2–3, NHMUK 1903.7.1.3147, NZSI M.6142, NZSI M.6143	Sibbum, Abor Hills	NZSI “No. 6142–43” (2 shells)	Valid lectotype and paralectotype designation by Sutcharit et al. (2019), while contradicting the original intension of onomatophore designation by Godwin-Austen (1915).
15. *Theobaldius oakesi*	Lectotype (design. nov.) NHMUK 1903.7.1.3083/1 (Fig. 8B).	Tsanspu Valley, Abor Hills	NHMUK 1903.7.1.3083 (2 shells)	–
	Paralectotype NHMUK 1903.7.1.3083/2 (Fig. 8C).			

### 1. Cyclophorus aborensis

Taxon classificationAnimaliaPolypodialesPolypodiaceae

Godwin-Austen, 1915

C2E02180-D868-56AB-8087-C37E3A6EE9C5

Cyclophorus aborensis Godwin-Austen, 1915: 494, pl. 38, fig. 1, 1a–c. Nantarat et al. 2014a: 3, 4, fig. 2a, b.

#### Type material.

***Holotype*** NZSI M.6010/1. ***Paratypes*** NZSI M.6009/1 (1 shell) from Rotung, 2000 ft., near Egar stream; NHMUK 1903.7.1.3048 (2 shells; Nantarat et al. 2014a; fig. 2b) from Kalek; NHMUK 1903.7.1.3051 (1 shell; Nantarat et al. 2014a; fig. 2a) from Renging.

#### Other non-type materials.

NHMUK 1903.7.1.3049 (2 shells) from Rami Dambang, Abor.

**Figure 7.**
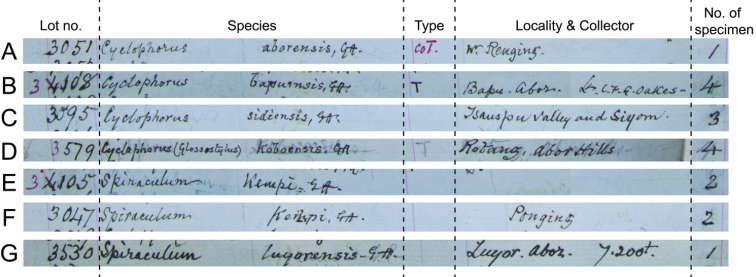
Record in the NHM Register of Godwin-Austen Collection in Godwin-Austen’s handwriting of **A** No. 3051 *Cyclophorus aborensis***B** No. 3108 Cyclophorus (Glossostylus) bapuensis**C** No. 3095 Cyclophorus (Glossostylus) sidiensis**D** No. 3579 Cyclophorus (Glossostylus) koboensis**E** No. 3105 *Spiraculum kempi***F** No. 3047 *Spiraculum kempi*, and **G** No. 3530 *Spiraculum luyorensis*.

Specimen “No. 6010/1 in Ind. Mus.” (NZSI M.6010/1) is deemed the holotype fixed by original designation as explained above. All specimens in the remaining lots are paratypes, except for lot “No. 3049 B.M.”, which Godwin-Austen (1915) regarded as a dwarf variety. In addition, the designation of a lectotype from lot NHMUK 1903.7.1.3051, labelled with “Co-Type” in Godwin-Austen’s handwriting (Fig. 6A) and recorded as “CoT” in the Register of Godwin-Austen (Fig. 7A), by Nantarat et al. (2014a) is invalid. The type locality of this taxon is restricted to “Rotung, 2000 ft., near Egar stream” only.

### 2. Cyclophorus (Glossostylus) bapuensis

Taxon classificationAnimaliaPolypodialesPolypodiaceae

Godwin-Austen, 1915

3A915DD8-EACE-53FF-8270-867A7A571290

Cyclophorus (Glossostylus) bapuensis Godwin-Austen, 1915: 494, 495, pl. 38, fig. 2, 2a–c. Nantarat et al. 2014a: 6, fig. 4a, b.

#### Type material.

***Lectotype*** NHMUK 1903.7.1.3108/1 (Nantarat et al. 2014a: fig. 4a). ***Paralectotypes*** NHMUK 1903.7.1.3108/2–3 (2 shells; Nantarat et al. 2014a: fig. 4b) from Abor Hills, vicinity of Bapu.

Only one specimen lot, “No. 3108 Brit. Mus.”, was mentioned in the “Type” paragraph of the original description and was labelled as “Type” in Godwin-Austen’s handwriting (Nantarat et al. 2014a: fig. 1a). One figured specimen from this lot was marked as “Type” in the plate caption. As there are three specimens in type lot NHMUK 1903.7.1.3108, Nantarat et al. (2014a) designated a lectotype (NHMUK 1903.7.1.3108/1; Nantarat et al. 2014a: fig. 4a) based on the specimen figured in the original description, corresponding to the given shell measurements. This lectotype designation is here confirmed as valid. The record in the Register of the Godwin-Austen Collection reveals that there were originally four specimens in this lot (Fig. 7B).

### 3. Cyclophorus (Glossostylus) sidiensis

Taxon classificationAnimaliaPolypodialesPolypodiaceae

Godwin-Austen, 1915

33CB1BD5-1AC4-5DF0-B40B-1816F378B652

Cyclophorus (Glossostylus) sidiensis Godwin-Austen, 1915: 495, pl. 38, fig. 3, 3a–c.

#### Type material.

***Syntypes*** NZSI M.6002 from Sidi River, Abor Hills; NZSI M.6001 from Rotung; NHMUK 1903.7.1.3095 (3 shells) from Tsanpu Valley.

The type series of this species comprises three specimen lots. Without any explicit holotype designation or equivalent expression, all specimens in these three lots are syntypes. However, only specimen lot “No. 6002 Ind. Mus.” was mentioned in the “Type” paragraph, and one specimen from this lot was figured with the annotation “Type” in the plate caption. This specimen lot is, therefore, the onomatophore as originally intended by Godwin-Austen (1915). Subsequent authors should select that particular figured specimen from lot NZSI M.6002 as the lectotype. There is one additional specimen lot (NHMUK 1903.7.1.3095; Fig. 7C) that contains syntypes, but currently this lot could not be located in the NHM.

### 4. Cyclophorus (Glossostylus) koboensis

Taxon classificationAnimaliaPolypodialesPolypodiaceae

Godwin-Austen, 1915

D25410F2-0B4C-5B75-9A39-17AFD0F4B716

Cyclophorus (Glossostylus) koboensis Godwin-Austen, 1915: 495, 496, pl. 38, fig. 4, 4a–d. Nantarat et al. 2014a: 14, fig. 12a, b. Sajan et al. 2019: 25–28, fig. 1.

#### Type material.

***Holotype*** NZSI M.6015/1 (Sajan et al. 2019: fig. 1). ***Paratypes*** NZSI M.6019–20 (2 shells) from Rotung; NHMUK 1903.7.1.3045 (3 shells) from Ponging; NHMUK 1903.7.1.3117 (2 shells) from Yamme Valley; NHMUK 1903.7.1.3579 (4 shells; Nantarat et al. 2014a: fig. 12a, b) from Rotung.

#### Other non-type materials.

NHMUK 1903.7.1.3581 (1 shell) from between N. 28°15'–29°15'L. 94°50'–95°10'.

Specimen “No. 6015 Ind. Mus.” (NZSI M.6015/1) is deemed the holotype fixed by original designation as explained above. All specimens in the remaining lots are paratypes, except for lot “No. 3581 B.M.” of which Godwin-Austen (1915) stated that it “comes very close to this spices [sic; species]”. We regard this as a doubtful attribution (ICZN 1999: Art. 72.4.1). The status of the type series and of the holotype has been correctly clarified by Sajan et al. (2019), whereas the designation of a lectotype and paralectotypes from lot NHMUK 1903.7.1.3579, labelled with “Co-Type” in Godwin-Austen’s handwriting (Fig. 6B) and recorded as “CoT” in the Register of Godwin-Austen (Fig. 7D), by Nantarat et al. (2014a) is invalid. The type locality of this taxon is retained and restricted to “Abor Hills, Kobo, on right bank of Tsanspu or Brahmaputra River” only.

### 5. Spiraculum oakesi

Taxon classificationAnimaliaArchitaenioglossaCyclophoridae

Godwin-Austen, 1915

5EE8EA5D-A280-5B4C-BF1A-78F2051B074E

Spiraculum oakesi Godwin-Austen, 1915: 496, pl. 39, fig. 3, 3a.
Pearsonia oakesi – Sutcharit et al. 2019: 43, fig. 10d, e.

#### Type material.

***Lectotype*** (design. nov.) NHMUK 1903.7.1.3081/1 (Sutcharit et al. 2019: fig. 10d). ***Paralectotypes*** NHMUK 1903.7.1.3081/2–5 (4 shells; Sutcharit et al. 2019: fig. 10e) from Abor Hills; NZSI (2 shells).

The type series of this species comprises lot “No. 3081 Brit. Mus.” and two uncatalogued specimens in the Indian Museum. Without any explicit holotype designation or equivalent expression, all specimens are syntypes. However, only specimen lot “No. 3081 Brit. Mus.” was mentioned in the “Type” paragraph, and one specimen from this lot was figured with the annotation “Type” in the plate caption. The author explicitly indicated that five specimens were examined, and type lot NHMUK 1903.7.1.3081 accordingly contains five specimens with a label in Godwin-Austen’s handwriting stating “Type”. We hereby designate the specimen from lot NHMUK 1903.7.1.3081 which is figured in the original description and in Sutcharit et al. (2019: fig. 10d) as the lectotype (NHMUK 1903.7.1.3081/1) to stabilize the name. This lectotype designation is based on the idea that Godwin-Austen (1915) selected this specimen lot as onomatophores.

### 6. Spiraculum kempi

Taxon classificationAnimaliaArchitaenioglossaCyclophoridae

Godwin-Austen, 1915

E0C0186F-ED08-500C-B43A-8B4882701129

Spiraculum kempi Godwin-Austen, 1915: 496, 497, pl. 39, figs 4, 4a, 5, 5a.
Pearsonia kempi – Sutcharit et al. 2019: 31, fig. 7d, e.

#### Type material.

***Lectotype*** (design. nov.) NHMUK 1903.7.1.3105/1 (Sutcharit et al. 2019: fig. 7d). ***Paralectotypes*** NHMUK 1903.7.1.3105/2 (1 shell) from Abor Hills; NHMUK 1903.7.1.3047 (2 shells; Sutcharit et al. 2019: fig. 7e) from Ponging; NZSI (2 shells).

The type series of this species comprises two specimen lots, “No. 3105 Brit. Mus.” and “No. 3047 B.M.”, and two uncatalogued specimens in the Indian Museum. Without any explicit holotype designation or equivalent expression, and given that the Register of Godwin-Austen Collection explicitly states that each lot contains two specimens (Fig. 7E, F), all specimens in these lots are syntypes. However, only the figured specimen from lot “No. 3105 Brit. Mus.” from Abor Hills was marked as “Type” in the plate caption (Fig. 1B) and this lot is labelled as “Type” in Godwin-Austen’s handwriting (Fig. 4A). In contrast, the figured specimen from lot “No. 3047 B.M.” from Ponging was not marked as “Type” in the plate caption and this lot is labelled as “Typic” in Godwin-Austen’s handwriting (Fig. 4B). Although, according to Art. 73.2., all the specimens of the type series are automatically syntypes and have equal status in being name-bearing types, we hereby designate the specimen from lot NHMUK 1903.7.1.3105 that is figured in Godwin-Austen (1915: pl. 39, fig. 4, 4a) and Sutcharit et al. (2019: fig. 7d) as the lectotype (NHMUK 1903.7.1.3105/1) to stabilize the name. This lectotype designation is based on the idea that Godwin-Austen (1915) selected this specimen lot as onomatophores and thus prevents any future attempt to designate a specimen from the other lots as the lectotype. The type locality of this taxon is restricted to “Abor Hills”.

### 7. Spiraculum planum

Taxon classificationAnimaliaArchitaenioglossaCyclophoridae

Godwin-Austen, 1915

61D810B1-8000-5D9A-80FF-8D4D19EA3C34

Spiraculum planum Godwin-Austen, 1915: 497, pl. 39, fig. 6, 6a, b.

#### Type material.

***Syntypes*** NZSI M.5992 from Upper Rotung, Abor Hills; NZSI M.5992a from Yembung.

#### Other non-type materials.

NHMUK 1903.7.1.3596 (1 shell) from the Miri Hills.

The type series of this species comprises two specimen lots, except for lot “No. 3596 B.M.” that Godwin-Austen (1915) regarded as a small variety. Without any explicit holotype designation or equivalent expression, all specimens in these lots are syntypes. However, only specimen lot “No. 5992 Ind. Mus.” was mentioned in the “Type” paragraph, and one specimen from this lot was figured with the annotation “Type” in the plate caption. This specimen lot is, therefore, deemed onomatophore as originally intended by Godwin-Austen (1915). Therefore, subsequent authors should select that particular figured specimen from the lot NZSI M.5992 as the lectotype.

### 8. Pterocyclos aborensis

Taxon classificationAnimaliaArchitaenioglossaCyclophoridae

Godwin-Austen, 1915

8D9A3C3B-C41F-54A4-8FFE-0DCE790BA2FB

Pterocyclos aborensis Godwin-Austen, 1915: 498, pl. 39, fig. 1, 1a. Sutcharit et al. 2019: 5, fig. 1b, c.

#### Type material.

***Lectotype*** (design. nov.) NHMUK 1903.7.1.3104/1 (Sutcharit et al. 2019: fig. 1b). ***Paralectotypes*** NHMUK 1903.7.1.3104/2–3 (2 shells; Sutcharit et al. 2019: fig. 1c) from Abor Hills; NHMUK 1903.7.1.3046 (2 shells) from Ponging; NHMUK 1903.7.1.3050 (3 shells) from Rami Lambang.

The type series of this species comprises three specimen lots. Without any explicit holotype designation or equivalent expression, all specimens in these lots are syntypes. However, only specimen lot “No. 3104 Brit. Mus.” from Abor Hills was mentioned in the “Type” paragraph, one figured specimen from this lot was marked as “Type” in the plate caption, and this lot was labelled as “Type” in Godwin-Austen’s handwriting (Fig. 5A). In contrast, two remaining lots, “No. 3046 B.M.” from Ponging and “No. 3050 B.M.” from Rami Lampang, were not labelled as type (Fig. 5B, C). Although, according to Art. 73.2., all the specimens of the type series are automatically syntypes and have equal status in being name-bearing type, we hereby designate the specimen from lot NHMUK 1903.7.1.3104 that is figured in the original description and in Sutcharit et al. (2019: fig. 1b) as the lectotype (NHMUK 1903.7.1.3104/1) to stabilize the name. This lectotype designation is based on the idea that Godwin-Austen (1915) selected this specimen lot as onomatophores and thus prevents any future attempt to designate a specimen from the other lots as the lectotype. The type locality of this taxon is restricted to “Abor Hills”.

### 9. Pterocyclos miriensis

Taxon classificationAnimaliaArchitaenioglossaCyclophoridae

Godwin-Austen, 1915

D5C51198-9E2A-5266-A247-6B2D88424DAD

Fig. 8A

Pterocyclos miriensis Godwin-Austen, 1915: 498, pl. 39, fig. 2, 2a–c.

#### Type material.

**Figure 8.**
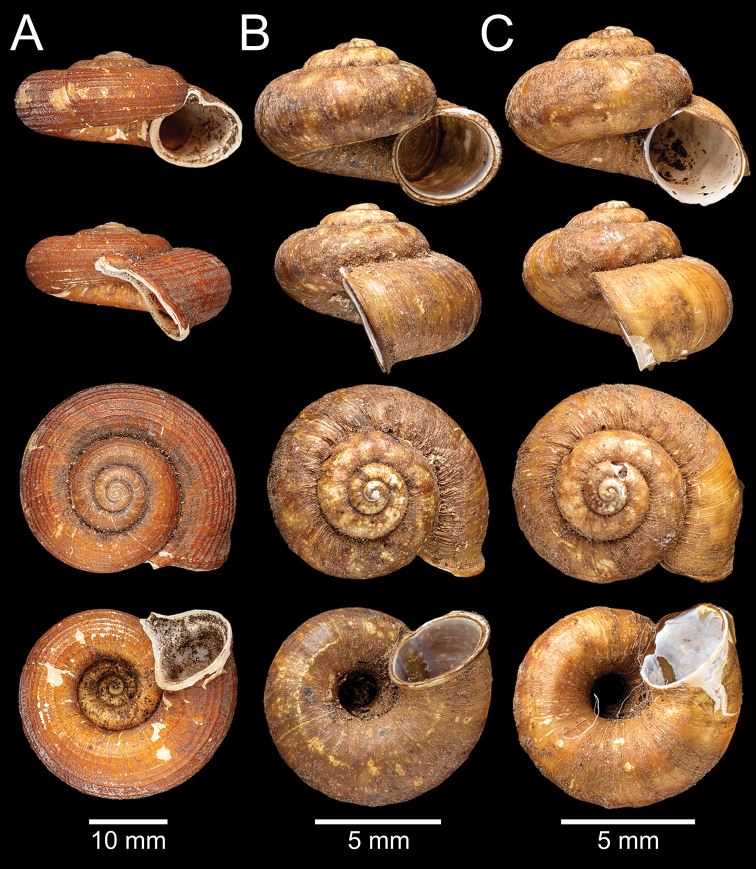
**A** lectotype of *Pterocyclos miriensis*, NHMUK 1903.7.1.3580/1 **B, C***Theobaldius oakesi*: **B** lectotype NHMUK 1903.7.1.3083/1 and **C** paralectotype NHMUK 1903.7.1.3083/2.

***Lectotype*** (design. nov.) NHMUK 1903.7.1.3580/1 (Fig. 8A). ***Paralectotypes*** NHMUK 1903.7.1.3580/2–4 (3 shells) from Miri Hills.

Godwin-Austen (1915) explicitly stated that four specimens of this taxon were obtained, the type specimen lot number was given as “No. 3580 Brit. Mus.” and three specimens were transferred to the Indian Museum. However, currently there are four specimens in lot NHMUK 1903.7.1.3580, so it is presumed that none were sent to the NZSI. Without any explicit holotype designation or equivalent expression, these four specimens are syntypes. The specimen figured in the original description that corresponds to the shell measurements given is hereby designated as the lectotype (NHMUK 1903.7.1.3580/1; Fig. 8A) to stabilize the name.

### 10. Pterocyclos spiramentum

Taxon classificationAnimaliaArchitaenioglossaCyclophoridae

Godwin-Austen, 1915

CE63F534-5027-51AA-8246-F488BB5A7374

Pterocyclos spiramentum Godwin-Austen, 1915: 498, 499, pl. 40, fig. 4, 4a, b. Sutcharit et al. 2019: 49, fig. 11j.

#### Type material.

***Holotype*** NHMUK 1903.7.1.3082 (Sutcharit et al. 2019: fig. 11j).

Godwin-Austen (1915) explicitly stated that only one specimen was obtained and belonged to specimen lot “No. 3082 Brit. Mus.” According to Art. 73.1.2., Sutcharit et al. (2019) validly deemed this specimen (NHMUK 1903.7.1.3082) as the holotype fixed by monotypy.

### 11. Pterocyclos brahmakundensis

Taxon classificationAnimaliaArchitaenioglossaCyclophoridae

Godwin-Austen, 1915

74AF369B-DF81-5713-9D81-A8AE59CEF223

Pterocyclos brahmakundensis Godwin-Austen, 1915: 499, 500, with text figure 1. Sutcharit et al. 2019: 14, fig. 3d, e.

#### Type material.

***Lectotype*** (design. nov.) NHMUK 1903.7.1.713/1 (Sutcharit et al. 2019: fig. 3d). ***Paralectotypes*** NHMUK 1903.7.1.713/2–3 (2 shells; Sutcharit et al. 2019: fig. 3e) from Brahmakund, Eastern Assam.

Godwin-Austen (1915) explicitly stated that three specimens of this taxon were obtained, and the type specimen lot number was “No. 713 B.M.” Without any explicit holotype designation or equivalent expression, these three specimens are syntypes. The specimen figured in Sutcharit et al. (2019: fig. 3d) that corresponds to the text figure and the shell measurements given in the original description is hereby designated as the lectotype (NHMUK 1903.7.1.713/1) to stabilize the name.

### 12. Spiraculum luyorensis

Taxon classificationAnimaliaArchitaenioglossaCyclophoridae

Godwin-Austen, 1915

1C0FB8D3-8875-57C2-91CF-54C5D98BCB26

? Spiraculum luyorensis Godwin-Austen, 1915: 500, pl. 40, fig. 5, 5a, b.
Pearsonia luyorensis – Sutcharit et al. 2019: 36, fig. 8d.

#### Type material.

***Lectotype*** (design. nov.) NHMUK 1903.7.1.3530/1 (Sutcharit et al. 2019: fig. 8d).

Only one specimen lot, “No. 3530 Brit. Mus.”, was mentioned in the original description and associated with the “Type” paragraph. Although this type lot contains only one specimen and the Register of Godwin-Austen Collection reveals that there is only one specimen in this lot (Fig. 7G), it is nevertheless not evident in the original description that this taxon is based on a single specimen (ICZN 1999: Art. 73.1.2). Therefore, without any explicit holotype designation or equivalent expression, Sutcharit et al. (2019) validly deemed this specimen (NHMUK 1903.7.1.3530) as a syntype, following Recommendation 73F. We hereby designate this specimen, which is figured in the original description and also figured in Sutcharit et al. (2019: fig. 8d), as the lectotype (NHMUK 1903.7.1.3530/1) to stabilize the name.

### 13. Spiraculum putaoensis

Taxon classificationAnimaliaArchitaenioglossaCyclophoridae

Godwin-Austen, 1915

BA410278-04B4-54C9-AD3B-2824B47173FD

Spiraculum putaoensis Godwin-Austen, 1915: 500, 501, pl. 40, fig. 3, 3a, b.
Pearsonia putaoensis – Sutcharit et al. 2019: 46, 48, fig. 10j, k.

#### Type material.

***Lectotype*** (design. nov.) NHMUK 1903.7.1.3598/1 (Sutcharit et al. 2019: fig. 10j). ***Paralectotypes*** NHMUK 1903.7.1.3598/2–3 (2 shells; Sutcharit et al. 2019: fig. 10k) from Putao, Upper Burma.

Godwin-Austen (1915) explicitly stated that three specimens of this taxon were obtained but did not specify the specimen lot number to which these specimens belong. Without any explicit holotype designation or equivalent expression, these three specimens in lot NHMUK 1903.7.1.3598 labelled as “Type” in Godwin-Austen’s handwriting are syntypes. The specimen figured in Sutcharit et al. (2019: fig. 10j) that is closest to the given shell measurements and figured in the original description is hereby designated as the lectotype (NHMUK 1903.7.1.3598/1) to stabilize the name.

### 14. Spiraculum minimum

Taxon classificationAnimaliaArchitaenioglossaCyclophoridae

Godwin-Austen, 1915

A2B93CCA-A2AF-5243-8C8F-1FF9671A66E4

Spiraculum minimum Godwin-Austen, 1915: 501, 502, pl. 40, fig. 2, 2a–c.
Pearsonia minima – Sutcharit et al. 2019: 40, fig. 9b, c.

#### Type material.

***Lectotype*** NHMUK 1903.7.1.3145/1 (Sutcharit et al. 2019: fig. 9b). ***Paralectotypes*** NHMUK 1903.7.1.3145/2–3 (2 shells; Sutcharit et al. 2019: fig. 9c) and NHMUK 1903.7.1.3147 (2 shells) from Sibbum, Abor Hills; NZSI M.6142 (1 shell) and NZSI M.6143 (1 shell) from Jeku, Abor Hills.

The type series of this species comprises four specimen lots. Only two specimen lots, “No. 6142–43 Ind. Mus.”, which were explicitly stated to contain two specimens from “Jeku, Abor Hills”, were mentioned in the “Type” paragraph, and one of these two specimens was figured and marked as “Type” in the plate caption. In contrast, the remaining lots were mentioned in the body of the text of subsequent paragraphs. This could mean that Godwin-Austen (1915) selected specimen lots “No. 6142–43 Ind. Mus.” as onomatophores. However, without any explicit holotype designation or equivalent expression, all specimens of the type series are automatically syntypes with equal status in being name-bearing types. Thus, the designation of the lectotype from lot NHMUK 1903.7.1.3145, labelled as “Typical” in Godwin-Austen’s handwriting (Fig. 6C), by Sutcharit et al. (2019) is valid under the Code, although this action contradicted the intention of the onomatophore designation by Godwin-Austen (1915). The type locality of this taxon is restricted to “Sibbum, Abor Hills”.

### 15. Theobaldius oakesi

Taxon classificationAnimaliaArchitaenioglossaCyclophoridae

(Godwin-Austen, 1915)

89975B81-358F-5504-A525-A9B965250ABB

Fig. 8B, C

Cyclophorus oakesi Godwin-Austen, 1915: 502, pl. 40, fig. 1, 1a.
Theobaldius oakesi – Gude 1921: 39. Ramakrishna and Dey 2010: 44.

#### Type material.

***Lectotype*** (design. nov.) NHMUK 1903.7.1.3083/1 (Fig. 8B). ***Paralectotype*** NHMUK 1903.7.1.3083/2 (1 shell; Fig. 8C) from Tsanspu Valley, Abor Hills.

Godwin-Austen (1915) explicitly stated that two specimens of this taxon were obtained, with a type specimen lot number of “No. 3083 Brit. Mus.”. Without any explicit holotype designation or equivalent expression, both specimens are syntypes. The specimen figured in the original description that corresponds to the shell measurements given is hereby designated as the lectotype (NHMUK 1903.7.1.3083/1; Fig. 8B) to stabilize the name.

## Supplementary Material

XML Treatment for Cyclophorus aborensis

XML Treatment for Cyclophorus (Glossostylus) bapuensis

XML Treatment for Cyclophorus (Glossostylus) sidiensis

XML Treatment for Cyclophorus (Glossostylus) koboensis

XML Treatment for Spiraculum oakesi

XML Treatment for Spiraculum kempi

XML Treatment for Spiraculum planum

XML Treatment for Pterocyclos aborensis

XML Treatment for Pterocyclos miriensis

XML Treatment for Pterocyclos spiramentum

XML Treatment for Pterocyclos brahmakundensis

XML Treatment for Spiraculum luyorensis

XML Treatment for Spiraculum putaoensis

XML Treatment for Spiraculum minimum

XML Treatment for Theobaldius oakesi
